# Effects of Educational Interventions on Dietary Adherence among Type 2 Diabetics in Zahedan: Using the Health Action Process Approach

**DOI:** 10.4314/ejhs.v33i4.3

**Published:** 2023-07

**Authors:** Sara Moghimi, Abolfazl Payandeh, Soheila Ranjbaran, Maryam seraji

**Affiliations:** 1 MSc student of Health Education and Health Promotion, Student Research Committee, Zahedan University of Medical Sciences, Zahedan, Iran; 2 Assistant Professor, Department of Statistics, Infectious and Tropical Diseases Research Center, Zahedan University of Medical Sciences, Zahedan, Iran; 3 Department of Public Health, Sarab Faculty of Medical Sciences, Sarab, Iran; 4 Assistant Professor, Health Promotion Research Center, Zahedan University of Medical Sciences, Zahedan, Iran

**Keywords:** Health Action Process Approach, Dietary Adherence, Type 2 Diabetes

## Abstract

**Background:**

Type 2 diabetes is the most common type of diabetes, and dietary adherence is a self-care practice. This research aims to improve dietary adherence among type 2 diabetics in Zahedan using the HAPA model.

**Methods:**

In this cross-sectional study, a total of 210 type 2 diabetics admitted to hospital clinics in Zahedan during summer 2022 were selected. The intervention group (n = 105) and the control group (n = 105) were from hospitals in Zahedan. Samples were selected by the simple random sampling method among the diabetics. After data collection using the demographic characteristics questionnaire and the Dietary Adherence Questionnaire and the HAPA model constructs questionnaire, the pre-test analysis was performed. One and three months after the educational intervention, the questionnaires on HAPA model constructs and self-care behavior were filled out by the patients. Next, data were analyzed using independent t-test, chi-square test, and the Shapiro-Wilk test in SPSS 23.

**Results:**

The results showed that all of the HAPA model constructs had significant differences, one and three months after the educational intervention (P = 0.001), indicating the effectiveness of education in the intervention group. However, there was no significant difference in the control group (P = 0.009).

**Conclusion:**

After the intervention using the HAPA model, the model's constructs had a significant impact on the patients' self-care of dietary adherence following the training.

## Introduction

Diabetes is one of the most common chronic and progressive diseases (1), as well as a metabolic disorder characterized by hyperglycemia caused by impaired insulin secretion, defective insulin action, or both (2, 3). Diabetes is considered the leading cause of death worldwide, especially in developing countries (4, 5). According to the most recent report presented by the International Diabetes Federation, the global prevalence of diabetes will reach 700 million people (12.2%) by 2045 (5, 6). According to a report published by the World Health Organization in 2018, at least 10% of Iranians over 18 will have high blood glucose by 2045, which is estimated to reach 13.9% (4). Type 2 diabetes imposes a global health burden with a great impact on individuals, families, and society (7). Type 2 diabetes is associated with complications, such as cardiovascular diseases (CVDs), retinopathy, nephropathy, cancer, increased risks of premature death, glaucoma, cataracts, foot problems, skin infections, and urinary tract infections (1, 8). In fact, risk factors for type 2 diabetes include smoking, alcohol consumption, unhealthy diets, obesity, physical inactivity, and family history (9). About 80% of the complications of type 2 diabetes can be prevented by reducing modifiable risk factors through self-care behaviors (10).

The World Health Organization has recommended the management and self-care of diabetes as indicators of the coverage of essential health services (4). Self-care is one of the main objectives of the treatment and prevention of type 2 diabetes complications (2). Diabetes self-care includes daily activities that people must perform to control or reduce the impact of the disease on their health and wellbeing so as to prevent further complications of the disease (2). Self-care behaviors, such as weight loss, medication use, physical activity, and healthy eating can reduce the risk of diabetes by 58% (10, 11). Showing positive self-care behavior is essential for achieving diabetes treatment goals and maximizing the quality of life (12). Adherence to a healthy diabetic diet is the key to displaying healthy behavior (13). Fruit and vegetable intake is an indicator of having a healthy diet (14). Educational interventions meant to bring about desirable behavioral changes will be designed more effectively if all determinants of the target behavior are considered. Behavior change theories help provide a better insight into the factors affecting behavior in the target population and help choose an appropriate approach to designing, implementing, and evaluating interventions (1). The Health Action Process Approach (HAPA) suggests that the adoption, initiation, and maintenance of health behaviors should be understood as a structured process that includes motivation and volition phases (13). The motivational phase includes risk perception, outcome expectancies, and action self-efficacy, which leads to a behavioral intention (15). On the other side, the volition phase includes coping self-efficacy, recovery self-efficacy, action planning, and coping planning, which leads to actual health behavior, being applied to bridge the gap between intentions and behaviors (15). In this phase, the change must be planned, initiated, and maintained, with relapses needing to be managed. A plan is usually a set of concrete ideas about when, where, and how to act on the produced intention (16).

Research on the HAPA in Iran shows that seven constructs of the HAPA are effective in determining a healthy diet for diabetics, explaining 81.1% of the total variance (17). In Australia, MacPhail. et al. reported that the HAPA was effective in predicting health outcomes in type 2 diabetes patients (18), but it did not improve healthy eating. Given the sociocultural conditions of Zahedan and that diabetes clinics in Zahedan were investigated in a pilot evaluation (19), the diabetic patients did not have enough knowledge of diabetic diet adherence. Moreover, due to the large number of patients visiting clinics, endocrinologists and healthcare providers working in clinics did not have enough time for instructing the patients. The present study aimed to evaluate the effects of educational interventions on dietary adherence among type 2 diabetics in two selected clinics in Zahedan, using the HAPA.

## Methods

**Study design and participants**: This quasi-experimental intervention study was conducted with a control group on 188 patients with type 2 diabetes, who were admitted to the diabetes clinics of Bu Ali and Khatam al-Anbiya (PBUH) Hospitals in Zahedan in 2022. Taking into account a 10% dropout rate, a total of 210 patients with type 2 diabetes were selected (105 in the intervention group and 105 in the control group). The intervention (diabetes clinic of Bu Ali Hospital) and control (diabetes clinic of Khatam al-Anbiya Hospital) clinics were chosen at random by coin toss. The inclusion criteria of the study included being less than 65 years old, being covered by Zahedan University of Medical Sciences with one's disease confirmed by a specialist, having passed six months since the diagnosis of one's disease, having a dietary adherence score of less than 3, and having completed the consent form for participation in the study. On the other side, the exclusion criteria included suffering from complications caused by diabetes, including kidney failure, blindness, amputation, cardiovascular diseases, cancer, mental illnesses, inability to understand the questions, not giving consent to participate in the study, and not attending more than one-third of the training sessions.

**Measures**: To attain the objectives of the research, the demographic questionnaire, the dietary adherence questionnaire, and the HAPA model constructs questionnaire were filled out by the patients meeting the inclusion criteria.

**Demographic information questionnaire**: This questionnaire included age, sex, marital status, level of education, occupation, and family income, along with questions about diabetes history, date of diabetes diagnosis, and the medication taken to control diabetes.

**Dietary adherence questionnaire**: The dietary adherence questionnaire for patients with type 2 diabetes was translated into Persian by Negarandeh. et al. to assess dietary adherence (20). In fact, it included nine questions, with seven four-choice questions (one point for each question) on a four-point Likert scale (never, rarely, sometimes, always), one two-point question (yes with one point and no with zero point), and one question about the days of the week the patient followed the diet. Scores lower than 3 meant low dietary adherence, 3-6 meant average dietary adherence, and greater than 6 meant high dietary adherence. The score range of this questionnaire was 0-9. Besides, the Cronbach's alpha coefficient for this study was confirmed at 0.85.

**The questionnaire about the constructs of the Health Action Process Approach model**: This questionnaire included 51 questions about the constructs of the HAPA model, which included behavioral intention, risk perception, outcome expectations, action self-efficacy, coping self-efficacy, recovery self-efficacy, action planning, coping planning, perceived barriers, and perceived benefits (13). The content validity ratio of CVR > 0.6 and content validity index of CVI > 0.7 for this questionnaire were obtained using a quantitative method, according to the Lawshe table (21). In addition, the average content validity index in this study for all questionnaire constructs was higher than 0.86 (21). Furthermore, the Cronbach's alpha calculation method was used to measure the internal consistency of the instrument, with the test-retest method employed to determine the reliability of the instrument in terms of repeatability.

**Figure 1:**
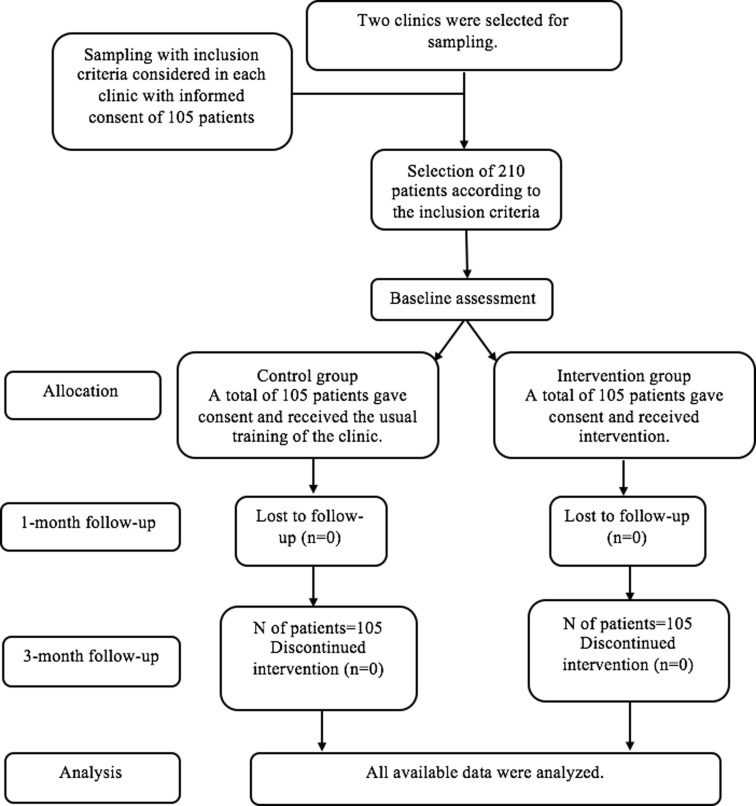
CONSORT trial flow chart

**Intervention**: The eligible subjects were selected through random sampling based on the list of the patients. After the complrtion of the dietary adherence questionnaire and the HAPA model constructs questionnaire by qualified patients, a pre-test analysis was performed. Next, four 40-minute educational intervention sessions were held in the form of lectures, educational booklets, and behavior self-report booklets. One and three months after the educational intervention, the HAPA model constructs questionnaire and the self-care behavior questionnaire were filled out by the patients. To meet ethical requirement in the research, at the end of the study, if the results were effective, atraining program would be provided to the control group (Figure 1).

**Statistical analysis**: After data collection, raw data were entered into the statistical software of SPSS 23. In addition, a chi-square test was used to compare proportions between the groups to check the assumption of independence. Besides, paired sample t-test was used to compare the average of quantitative variables within the group (before and after the intervention) if the assumptions of the parametric tests were fulfilled. To control the impact of possible and background confounding variables, apart from determining appropriate inclusion criteria and participation consent, regression models were used, if necessary.

**Ethics**: The ethics committee of Zahedan University of Medical Sciences approved this study under ethics code IR.ZAUMS.SPH.REC.1400.392. Before participating in the study, the participants were fully briefed on the research plan and objectives, and those willing to participate in the study signed the informed consent form.

## Results

This study included 210 diabetics with the mean age of 53.55 ± 8.27. In fact, the minimum and maximum age was 33 and 65 (p = 0.87), respectively. A total of 147 female patients (70%) and 63 male patients (30%) participated in this research (p = 0.88). The two selected hospitals were randomly assigned to the intervention and control groups by coin toss. The studied background variables were described and compared between the two groups, with the results reported. Accordingly, the results showed that the two groups had no statistically significant differences in terms of demographic variables, having been similar in this respect (p < 0/05). Furthermore, the results demonstrated that the variable of “duration of the patients' illness” did not follow a normal distribution pattern in either of the two groups (P < 0.05) (Table 1).

**Table 1:** Demographic characteristics of the participants (n=210)

Group→→		Intervention	Control	P-value
	
Demographic characteristics↓↓↓		Number (Percent)	Number (Percent)	
Gender*	**Male**	31 (29.5%)	32 (30.5%)	0.88*
	**Female**	74 (70.5%)	73 (69.5%)	
Age^**^		46.53	65.53	0.87^**^
Level of	Illiterate	37 (35.2%)	44 (41.9%)	0.8^*^
Education^*^	Elementary	27 (25.7%)	22(21%)	
	Guidance school	10 (9.5%)	8 (7.6%)	
	High school		20 (19%)	
	University degree	9 (8.6%)	11(10.5%)	
Marital status	Married	97 (92.4%)	94 (89.5%)	0.4^*^
	Widow(er)	7(6.7%)	7 (6.7%)	
	Divorced	1 (1%)	5 (2.4%)	
Employment Status	Unemployed	9 (8.6%)	14 (13.3%)	0.53^*^
	Employee	9 (8.6%)	10 (9.5%)	
	Business	7 (6.7%)	4 (3.8%)	
	Retired	13 (12.4%)	8 (7.6%)	
	Housewife	67 (63.8%)	69 (64.8%)	

**Associations between dietary adherence and the Health Action Process Approach**: The constructs of risk perception and action planning had a positive effect on self-care behaviors in terms of dietary adherence. Besides, coping self-efficacy had both indirect and direct effects (P < 0.001) (Table 2).

**Table 2:** Comparing self-care behavior and HAPA model constructs before, one month after, and three months after the intervention (n=210)

Time → Group↓		Before the intervention	One month after the intervention	Three months after the intervention	P-value
Dietary	Intervention	14.17±4.45	16.43±3.88	18.39±3.52	<0.001
adherence	Control	13.50 ± 4.75	13.50 ± 4.71	13.42 ± 4.77	
behavior ^⁕^	P-value	0.295	<0.001	<0.001	
Risk perception	Intervention	4.03 ± 2.14	5.72 ± 0.58	5.72 ± 0.58	<0.001
^⁕^	Control	4.12 ± 2.23	4.03 ± 2.21	4.03 ± 2.21	
	P-value	0.75	<0.001	<0.001	
Outcome	Intervention	9.22 ± 3.02	10.76 ± 1.76	11.62 ± 0.73	<0.001
expectancies^⁕^	Control	9.53 ± 3.03	9.00 ± 3.27	9.02 ± 3.42	
	P-value	0.47	<0.001	<0.001	
Behavioral	Intervention	6.26 ± 1.50	6.95 ± 1.03	7.87 ± 0.57	<0.001
intention^⁕^	Control	6.46 ± 1.50	6.44 ± 1.57	6.51 ± 1.52	
	P-value	0.335	0.005	<0.001	
Action	Intervention	4.89 ± 3.11	4.89 ± 3.11	8.50 ± 0.97	<0.001
planning^⁕^	Control	5.27 ± 3.23	4.75 ± 3.28	4.84 ± 3.24	
	P-value	0.365	0.763	<0.001	
Coping	Intervention	8.87 ± 3.92	11.83 ± 2.55	13.78 ± 1.45	<0.001
planning^⁕^	Control	10.43 ± 3.77	9.91 ± 3.90	10.05 ± 3.88	
	P-value	0.004	<0.001	<0.001	
Action self-	Intervention	8.00 ± 3.67	11.24 ± 2.53	13.67 ± 1.48	<0.001
efficacy^⁕^	Control	9.40 ± 4.01	9.10 ± 4.01	9.29 ± 3.86	
	P-value	0.009	<0.001	<0.001	
Coping self-	Intervention	9.50 ± 4.23	13.41 ± 2.84	15.70 ± 1.93	<0.001
efficacy^⁕^	Control	10.81 ± 4.49	10.02 ± 4.55	10.21 ± 4.45	
	P-value	0.031	<0.001	<0.001	
Recovery self-	Intervention	5.55 ± 2.69	7.28 ± 1.98	8.31 ± 1.25	<0.001
efficacy^⁕^	Control	4.97 ± 2.63	4.56 ± 2.63	4.60 ± 2.64	
	P-value	0.115	<0.001	<0.001	
Perceived	Intervention	17.10 ± 4.59	7.22 ± 3.37	3.80 ± 3.14	<0.001
barriers^⁕^	Control	19.94 ± 4.30	19.88 ± 4.15	19.70 ± 4.18	
	P-value	<0.001	<0.001	<0.001	
Perceived	Intervention	12.97 ± 2.72	15.18 ± 1.49	15.81 ± 0.62	<0.001
benefits^⁕^	Control	13.28 ± 2.68	13.38 ± 2.59	13.81 ± 2.69	
	P-value	0.415	<0.001	<0.001	

⁕ Parametric t-test

## Discussion

Diabetes is one of the major chronic diseases, with the prevention of its complications requiring lifestyle modifications. Against this backdrop, this study aimed to identify determinants of dietary adherence among patients with type 2 diabetes using the HAPA model in Zahedan, Iran.

The results of this study showed that a significant percentage of the patients did not follow a healthy diet pattern, which could have been due to their illiteracy, socioeconomic status of the studied community, and the lack of proper relevant planning for type 2 diabetes patients. Moreover, people with low levels of income and education did not adhere to their diets, having been consistent with other studies (13, 22).

Since the educational intervention in the current research was based on the HAPA model, relevant constructs were investigated. Accordingly, risk perception, one and three months after the intervention, showed a statistically significant difference between the experimental and control groups compared to the time before the intervention. In fact, changes in the intervention group were more significant in the first month than in the third month. This difference emanated from the fact that during the group training period, the patients understood the risks of not following the diet and realized consequent risks in the first month, having been provided with educational booklets and videos. Thus, they continued this trend until the end of the educational intervention. Other studies such as those of Bonner. (23), Bluo. et al. (24), and Zhou. et al. (25) were consistent with ours, showing an increase in risk perception after the intervention. In Schwarzer's study, the construct of risk perception had no significant relationship with behavioral intention, so it was not consistent with the present one (26). Risk perception is one of the factors affecting patients' intention; accordingly, the greater the risk perception is, the greater the intention to adhere to the diet will be.

Outcome expectations, one and three months after the intervention, showed a significant difference in the experimental group, having been due to the patients' knowledge of the outcomes of not following the diet during group training sessions and after they were given a checklist. In fact, outcome expectations are among the factors affecting patients' intentions. Research shows the importance of post-intervention outcome expectations (25, 27). However, the intervention in the study of Lippke. et al. did not increase outcome expectations. This was due to the fact that the questionnaires were completed by the patients themselves not at the presence of the researchers. Thus, it was not possible to fully monitor the way the forms were completed. Moreover, the intervention was performed five weeks after the intervention, having been different from the follow-up time of our research (28).

In the HAPA model, one and three months after the intervention, behavioral intentions in the test and control groups showed a statistically significant difference compared to the time before the intervention. Accordingly, after completing the educational program, it was observed that the lectures, questions, and answers designed to increase the patients' intention were able to increase their intention to follow the diet in the test group. Furthermore, the significance of risk perception and outcome expectancies contributed to the increase in patients' intention. Additionally, some studies showed that behavioral intention increased significantly after the intervention (13, 15, 29). Other studies were not consistent with the present one, which could have been due to differences in the target group and the length of the follow-up period for the constructs of the HAPA model (30, 31). The results of the present study showed that designing an educational program within the framework of the Health Action Process Approach could be more effective in increasing patients' intention to follow the diet.

The unique feature of the HAPA model is its consideration of various types of self-efficacy, including action self-efficacy, coping self-efficacy, and recovery self-efficacy (32). In fact, the improvement in the types of self-efficacy can lead to increased patient adherence to the diet. In view of the framework of the present study, action self-efficacy was one of the factors affecting patients' intention and increasing intention creation. In this study, a significant difference was observed in self-efficacy in the test group one and three months after the intervention. Likewise, other studies showed an increase in self-efficacy after the intervention (13, 33). The study of Miller. et al. was not consistent with the present one. This difference could be due to the fact that the patients who followed the diet in our study talked to other patients, which increased action self-efficacy (30).

Coping self-efficacy, one and three months after training in the intervention group, showed a significant difference with the control group. In fact, self-efficacy is essential in both stages of intention formation and behavior change. The results of the study by Ranjbaran, et al. (13) and some other studies showed the significance of coping self-efficacy after the intervention, having been consistent with our results (15, 16). However, the results of the studies by Ghisi, et al. (31) and Miller. et al. (30) were not consistent with those of the present study. This difference in the results could have been due to the difference in the target group of the study and the follow-up duration. In fact, recovery self-efficacy showed a statistically significant difference one and three months after training. This significant difference could be attributed to proper training, motivational text messages, and the instilling of the belief in patients that they could keep following a healthy diabetic diet even after consuming unhealthy foods for a period of time. Other studies were consistent with our results (13, 15). However, the study by Miller. et al. (30) was not consistent with ours. The reason for this inconsistency in the results could have been the length of the follow-up with the target group.

The volitional phase includes action planning and coping planning, playing a mediating role between intention and behavior (34). The present study showed that action planning, among other constructs of the HAPA model, was a better predictor of dietary adherence. In fact, people who planned in detail on why and how to follow their diet got better self-care results. In the same vein, other studies showed that action planning played a good mediating role between intention and behavior (13, 15, 25). Some studies reported the insignificance of action and coping planning three months after the intervention (30, 31).

Perceived barriers, in the test group, showed a significant difference one and three months after the intervention. In fact, the results showed the achievement of the objectives set regarding the barriers. Moreover, holding group classes, discussing problems related to diet non-adherence, and phone call follow-ups were effective in removing barriers, having been consistent with Ranjbaran, et al. (13, 15, 16) but inconsistent with Charkazy, et al. (35) and Rothman. et al. (36).

In the present study, perceived benefits one and three months after the intervention showed a significant increase compared to the time before the intervention in the intervention group. This has been due to the usefulness of the content of the educational booklets and text messages regarding the benefits of following the diet. In other studies, perceived benefits increased significantly after the intervention as well (37, 38).

Self-care behavior towards dietary adherence was significantly different between the two experimental and control groups one and three months after the intervention. In fact, self-care behavior did not change significantly in the control group, but there was a significantly increasing trend in the experimental group, having been consistent with the study of Welsh et al (39) and other similar studies (4, 40). However, the study of White. et al. (41) and another study were not consistent with our research in terms of dietary adherence (42).

Our study results showed that the intervention using the health action process approach model increased dietary adherence in patients with type 2 diabetes in Bu Ali and Khatam Al Anbia clinics in Zahedan. In developing such interventions, healthcare providers should specifically focus on following patients' diet.
